# A matter of time: how musical training affects time perception

**DOI:** 10.3389/fnins.2024.1364504

**Published:** 2024-04-29

**Authors:** Jahanvi Mittal, Khushi Kaur Juneja, Saumya Saumya, Anuj Shukla

**Affiliations:** Thapar School of Liberal Arts and Sciences, Thapar Institute of Engineering and Technology, Patiala, Punjab, India

**Keywords:** musical training, temporal bisection, time judgement, length of training, PSE

## Abstract

Musical training has been linked to changes in early attentional and perceptual processing. Thus, such an altered attentional and perceptual processing has enabled musicians to judge the duration differently than non-musicians. Although these claims seem intriguing, there are many questions that are not addressed yet, for example, how would the performance of musically-trained differ from that of untrained on visual and auditory temporal judgments? Is there any advantage to musically-trained person in temporal processing? To understand these questions, we thus conducted a series of Auditory and Visual Temporal Bisection Tasks on 32 musically-trained and 32 musically-untrained participants. We hypothesized that if music training modulates general sensitivity to temporal dimensions, then the temporal judgments of musically-trained participants would differ from those of untrained participants in both visual and auditory tasks. Each participant performed a total of 140 trials (70 visual and 70 auditory) in two different blocks. For each participant, a *Point of Subjective Equality* (PSE) was obtained for visual and auditory conditions. The findings revealed a significant modality effect on time perception, with auditory stimuli being consistently overestimated compared to visual stimuli. Surprisingly, the musically-trained group exhibited a tendency to underestimate duration relative to the musically-untrained participants. Although these results may appear counterintuitive at first glance, a detailed analysis indicates that the length of musical training plays a significant role in modulating temporal processing within the musically-trained group.

## Introduction

Time perception has been the subject of investigation for many years. Previous studies have reported that temporal perception can be influenced by various factors, including emotion (Droit-Volet et al., 2011, 2020), color (Thönes et al., 2018), magnitudes (Chang et al., 2011; Shukla and Bapi, 2021, 2022) intention (Wenke and Haggard, 2009; Makwana and Srinivasan, 2017; Donapati et al., 2024), and meditation (Singh and Srinivasan, 2019). In addition to these factors, several studies have suggested associations between certain abilities and the processing of temporal information (Perrone et al., 2023). For instance, meditation has been linked to a better temporal experience, with meditators tending to overestimate durations compared to non-meditators (Wittmann et al., 2015). These findings have been attributed to the enhanced attentional resources available to meditators, resulting in a more subjective experience of time for them. Similarly, previous literature suggests that ability related to rhythm influences our perception of time for example, music aptitude tests, such as the *Seashore Measures of Musical Talents* (Seashore et al., 1956), have shown that higher music aptitude scores are associated with improved performance on tasks involving the processing of temporal information. This connection between music ability and time perception is further supported by studies demonstrating that musicians exhibit superior abilities compared to non-musicians in detecting subtle time changes within auditory sequences (Jones and Yee, 1997).

The mechanism by which music influences temporal processing is associated with broader cognitive enhancements resulting from musical training. These cognitive improvements, such as attention and memory, subsequently impact the processing of time. For instance, research on musical training has demonstrated its positive effects on both visual and auditory processing (Moreno et al., 2014), potentially enhancing our visual and auditory abilities (Faßhauer et al., 2015). Additionally, numerous studies indicate that music training induces structural changes in the brain, specifically an increase in gray matter volume in motor, visuo-spatial, and auditory regions (Gaser and Schlaug, 2003) and thereby exhibiting improvement in these domains. Hyde et al. (2009) observed that children who underwent 15 months of music training outperformed control groups in motor and melody/rhythm tests, which was attributed to structural changes in specific motor and auditory areas. These structural changes were found to be associated with an individual’s musical proficiency and practice intensity. Moreover, musicians exhibit a larger superior temporal gyrus, involved in auditory processing, compared to non-musicians (Schneider et al., 2002), indicating potential superior performance on auditory time perception tasks. Findings from studies on neural plasticity further support this speculation, suggesting that music training benefits the neural processes engaged in processing temporal information. Furthermore, musicians demonstrate robust neural connections between auditory, somatosensory, and motor brain regions (Bangert and Altenmüller, 2003; Jäncke, 2009), which may enable them to achieve precise timing control with increased expertise.

Rammsayer and Altenmüller (2006) conducted a study to investigate differences in temporal processing in musicians and non-musicians. For this, 36 academically trained musicians and 36 non-musicians completed seven distinct auditory temporal tasks. Musicians demonstrated greater temporal acuity than non-musicians for auditory fusion, rhythm perception, and three different types of temporal discrimination tasks. Yet, their performance on temporal generalization tasks was comparable. Temporal generalizations, which require a sort of reference memory, appear to be unaffected by extensive music training, in contrast to the immediate online processing of temporal information in temporal discrimination tasks. Another study by Vibell et al. (2021) evaluated the effects of musical training to measure simultaneity perception and temporal acuity in three different conditions- Visual, Auditory, and Cross-modal conditions. The results indicated that musicians showed improved simultaneity perception and temporal acuity for visual discrimination and auditory discrimination tasks, respectively. In contrast to non-musicians, musicians exhibit selective improvements in temporal discrimination, which is possibly caused by greater attentional efficiency. These findings provide a first step in identifying the precise elements that are strengthened by musical expertise. However, this study used animal sounds as a stimulus. It is important to note that the different animal sounds may engage attention differently and thereby may have confounded the results. To minimize potential attentional variability arising from the stimulus feature, we used neutral sound tones as auditory stimuli in our experiment.

Keeping the previous findings in mind, it is evident that like other abilities such as meditation, musical ability is linked with temporal processing. It is important to note that previous studies examining the impact of musical training on temporal judgements have predominantly been done in the auditory domain and no studies to the best of our knowledge have explored this relation in the visual domain. Further, there is no clarity on how musical ability would affect temporal performance across modality. In other words, whether musical ability is limited to improved performance in auditory modality or can this lead to improved performance on the other modality such as visual. Therefore, the present study intends to explore the influence of musical ability within and between modalities. In order to study this, we took two different groups: musically-trained and musically-untrained. The groups were given Auditory and Visual Temporal Bisection Tasks. We hypothesized that if music training modulates general sensitivity to temporal dimensions, then the temporal judgments of musically-trained participants would differ from those of untrained participants in both visual and auditory tasks.

## Materials and methods

### Participants

For the purposes of our experiment, we collected data from two distinct groups: musically-trained participants and musically-untrained participants. The musically-trained group comprised individuals who had been actively engaged in music practice for a minimum of two years. We recruited a total of sixty-four volunteers (32 musically-trained and 32 musically-untrained with age range from 18 to 30 years) from Thapar Institute of Engineering and Technology, Patiala, Punjab, India, to participate in the two experiments (Visual and Auditory). Our sample size exceeded the minimum requirement of total of 36 participants, which was estimated using G*POWER 3 (Faul et al., 2007), in order to mitigate potential attrition due to the presence of outliers. As per the study design, we used the parameters: alpha level = 0.05, Power = 0.95, and effect size = 0.25 for the sample size estimation.

The experimental procedures and methodologies adhered to established guidelines and regulations, and the research received approval from the Institute Review Board (IRB) at Thapar Institute of Engineering and Technology, Patiala, Punjab, India. Informed consent was obtained from all participating volunteers, and none of them reported any visual or auditory impairments.

### Apparatus

The presentation and control of stimuli were administered using *OpenSesame* stimulus presentation software (Mathôt et al., 2012). The stimuli were displayed on a 17-inch CRT monitor (1024 × 768 resolution) operating at a frame rate of 100 Hz.

### Stimulus

All participants completed two experiments: the *Visual Temporal Bisection Task* and the *Auditory Temporal Bisection Task*. In the *Visual Temporal Bisection Task*, a black square measuring 2 degrees of visual angle was presented against a white background. On the other hand, in the *Auditory Temporal Bisection Task*, participants were presented with a sound tone which was constructed from a sine wave, had a duration of 1,000 ms and a frequency of 440 Hz. The auditory stimulus was delivered binaurally through *Sennheiser* headphones, with the sound volume being individually adjusted for each participant to ensure their comfort.

### Procedure

Participants were tested in a quiet room. They were asked to sit comfortably. The distance between the participant and the computer monitor was 57 cm. Participants were instructed to refrain from moving their heads during the experiment. Experimenters closely monitored the participants to ensure compliance with these instructions and to prevent any head movement throughout the experiments. The instruction was given to the participants individually in a verbal format. Each participant performed both Auditory and Visual Temporal Bisection Task. The order of the task was counterbalanced across the participants within each group (musically-trained and musically-untrained).

#### Experiment-1: Auditory Temporal Bisection Task

**FIGURE 1:**
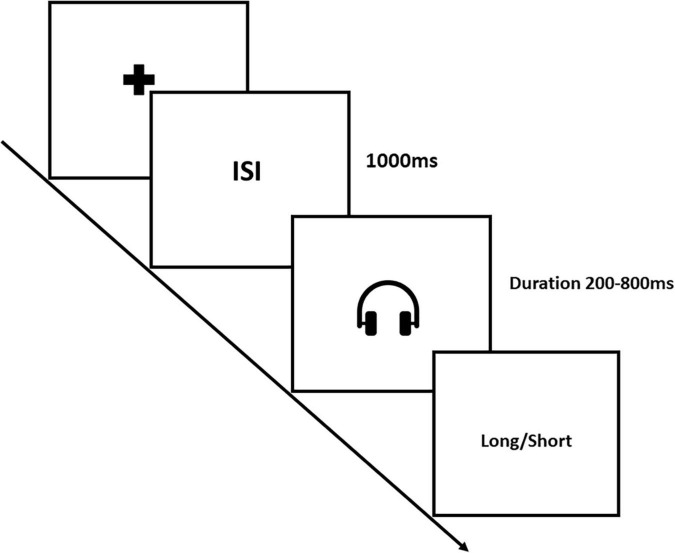
Illustrates the Auditory Temporal Bisection Task, with a specific focus on the testing phase. The trial started with the presentation of a fixation cross, followed by a blank screen serving as the inter-stimulus interval (ISI), lasting for 1,000 ms. Subsequently, a sound tone was presented aurally for probe durations ranging from 200 to 800 ms in a step of 100 ms. After the tone presentation, a blank screen appeared, prompting participants to record their response indicating whether the presented sound tone duration was perceived as closer to the long or short anchor duration.

The Auditory Temporal Bisection Task encompassed three distinct phases: Training, Feedback, and Testing. In the Training phase, participants were exposed to sound tones with durations of 200 and 800 ms, which served as the short and long anchor durations, respectively. To make sure that participants should get a sense of the long and short durations, they received 10 trials of short and 10 trials of long anchor durations aurally. Following the Training phase, participants proceeded to the Feedback phase. Here, sound tones of random durations, either 200 or 800 ms, were presented. Participants were asked to identify whether the tone was presented for the long anchor or the short anchor duration by pressing dedicated keys on the keyboard. Immediate feedback was provided on the computer screen, indicating whether their response was correct or incorrect. The goal of this phase was to ensure that participants achieved a 90% accuracy in their duration judgment task. Once this performance threshold was met, participants were taken to the subsequent Testing phase. In the Testing phase, sound tones were presented with probe durations ranging from 200 to 800 ms in increments of 100 ms. Participants were instructed to discriminate whether the presented tone duration was closer to the small anchor or the long anchor duration they had become familiar with during the Training phase. Their responses were recorded by pressing either the “L” key if the tone appeared closer to the long anchor duration or the “S” key if it seemed closer to the short anchor duration (see Figure 1). Each participant performed a total of 70 trials of auditory temporal judgements [7 (Durations: 200 to 800 ms) × 10 (Repetitions)].

#### Experiment-2: Visual Temporal Bisection Task

**FIGURE 2:**
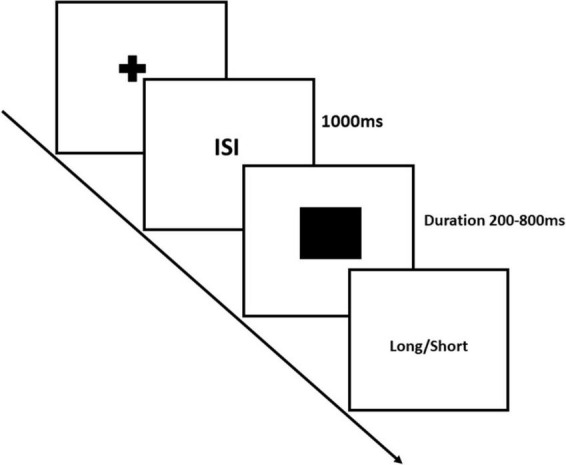
Illustrates the Visual Temporal Bisection Task, with a specific focus on the testing phase. The trial started with the presentation of a fixation cross, followed by a blank screen as the inter-stimulus interval (ISI), for 1,000 ms. Subsequently, a black square was displayed on the computer screen for probe durations ranging from 200 to 800 ms in a step of 100 ms. After the square presentation, a blank screen appeared, prompting participants to record their response indicating whether the presented square duration was perceived as closer to the long or short anchor duration.

In the Visual Temporal Bisection Task, the experimental procedures are identical to those of the Auditory Temporal Bisection Task, differing only in terms of the stimuli employed. Specifically, in the Visual Temporal Bisection Task, participants presented with a black square as the stimulus in all phases: training, feedback, and testing. The temporal durations presented in the Visual Temporal Bisection Task were consistent with those used in the Auditory Temporal Bisection Task (see Figure 2). Each participant performed a total of 70 trials of visual temporal judgements [7 (Durations: 200 to 800 ms) × 10 (Repetitions)]. Therefore, each participant performed a total of 140 trials [2 (Modality: Auditory and Visual) × 7 (Durations: 200 to 800 ms) × 10 (Repetitions)].

## Results

**FIGURE 3:**
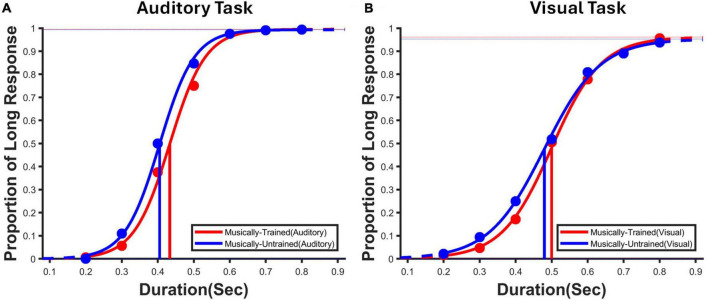
Average psychometric function for auditory and visual task conditions. **(A)** Psychometric fit for the auditory temporal judgments task, where participants presented the probe duration in the auditory modality for both musically-trained and musically-untrained participants. **(B)** Psychometric fit for the visual temporal judgments task, where participants presented the probe duration in the visual domain for both musically-trained and musically-untrained participants. Musically-trained participants performance is shown in red, whereas musically -untrained participants performance is indicated in blue.

The participants’ data were recorded in terms of long and short responses. We used psignifit-4, a MATLAB-based toolbox, and estimated a visual and auditory bisection point (BP) for each participant using a logistic function. The BP is the point at which 50% of the time participants would have perceived the presented duration to be closer to the short anchor and 50% of the time closer to the long anchor duration. The bisection point (BP) is also called point of subjective equality (PSE) and hereafter we use PSE instead of BP. Notably, a higher PSE value indicates an underestimation of duration, while a lower PSE value suggests an overestimation of duration. If the curve shows a leftward shift, it indicates overestimation, and a rightward shift shows an underestimation of duration (see Figure 3).

In order to see the modality as well as group effect on temporal perception, we compared the estimated PSEs of the participants across the modalities and the groups using a 2 (Modality: Auditory vs. Visual) X 2 (Group: musically-trained vs. musically-untrained) repeated measures mixed ANOVA, wherein *Modality* was a within-subject repeating factor, and the *Group* was a between-subject repeating factor. Based on previous research, we could assume that the participants would overestimate the duration in the auditory modality as compared to the visual one. There is also a hunch that musically-trained participants would relatively overestimate the duration of stimulus presented in the auditory modality as compared to the musically-untrained participants. The analysis yielded a significant main effect of modality [*F*(1, 62) = 108.32, *p* = 0.001, partial η^2^ = 0.636]. This suggests that the temporal processing for the auditory temporal judgments differs from the visual temporal judgments. Further, we also observed a significant difference for the group [*F*(1, 62) = 4.62, *p* = 0.036, partial η^2^ = 0.069]. This implies that the temporal processing in musically-trained and musically-untrained participants is different. However, we did not observe a significant interaction for Modality and Group [*F*(1, 62) = 0.271, *p* = 0.604, partial η^2^ = 0.004]. Further, an exploratory simple main effect analysis revealed a statistically significant difference in temporal processing for Auditory [*F*(1, 62) = 4.634, *p* = 0.035, partial η^2^ = 0.07] across different groups [Mean ± SD for musically-trained (0.435 ± 0.058) and musically-untrained (0.406 ± 0.050) group] but not for Visual Modality [*F*(1, 62) = 2.111, *p* = 0.151, partial η^2^ = 0.03] [Mean ± SD for musically-trained (0.511 ± 0.046) and musically-untrained (0.490 ± 0.067) group]. Further, to examine the magnitude of the null result observed in visual task condition, we used Bayesian independent *t*-test using *JASP 0.16.1* to test whether the PSE across the two groups significantly differed from one another. The Bayes factor analysis yields a value of BF_10_ = 0.621, considering that it is below one, we can conclude that there is favorable evidence for rejecting the alternative hypothesis. In other words, the results are 1.61 times more likely to have occurred under the null model (For tables, please refer to the Supplementary materials).

The previous findings suggest that the person with musical training overestimates the duration in auditory task than that of musically-untrained ones (Rammsayer and Altenmüller, 2006; Agrillo and Piffer, 2012). Therefore, it is important to note that we observed a counter intuitive result i.e., for the auditory task, musically-untrained participants overestimated the duration compared to musically-trained participants. These results are inconsistent with previous literature and led us to examine our musically-trained group from a different lens. On digging deeper within the musically-trained group we found that the years of training of the participants ranged from 2 to 14 years. We now wanted to investigate if this variability in the years of training is associated with the PSE for the auditory modality and thus conducted a Spearman’s rank correlation analysis for the same. The analysis revealed a negative correlation [*r*(32) = −0.418, *p* = 0.017] between length of training and PSE for auditory modality. This suggests that as the year of training increases the PSE for the auditory modality decreases, demonstrating the overestimation of the apparent duration. We also performed a correlation analysis to investigate the relationship between the length of training and PSE for the visual modality. In contrast to the auditory condition, the analysis revealed no significant correlation between the length of training and the PSE for the visual condition [*r*(32) = 0.006, *p* = 0.973].

Additionally, inspired by the correlation analysis, we segmented musically-trained participants into two subgroups based on their music training duration: those with less than 5 years training (13 participants) and those with more than 5 years of training (19 participants). Subsequently, we compared their PSE values for the auditory task. The independent *t*-test yielded a significant difference in PSE between participants with less than 5 years and those with more than 5 years of training [*t*(30) = −3.454, *p* = 0.002, Cohen’s *d* = −1.24]. Specifically, participants with over 5 years of training overestimated durations (0.409 ± 0.051) compared to those with less than 5 years of training (0.471 ± 0.048), consistent with prior research findings. Further, we conducted a similar analysis for the visual condition. Our results indicated no significant difference in PSE for the visual task between participants with less than 5 years (0.508 ± 0.047) and those with more than 5 years of training (0.513 ± 0.047) [*t*(30) = 0.267, *p* = 0.791, Cohen’s *d* = 0.096]. This suggests that the length of music training did not affect visual temporal processing (For tables, please refer to the Supplementary materials).

## Discussion

On reviewing the previous literature, it was found that musically-trained participants have enhanced temporal judgment than musically-untrained participants (Yee et al., 1994; Jones and Yee, 1997; Rammsayer and Altenmüller, 2006). However, studies conducted so far have examined this relationship predominantly on auditory modality. In the present study, we investigated how music training affects time perception using a temporal bisection task. More specifically, we examined whether the effect of musical training is limited to overestimation of an auditory stimulus or can this effect be transferred to the visual modality as well. The result of the present investigation suggests differential temporal processing for auditory stimuli in musically-trained participants compared to musically-untrained ones. However, no temporal processing differences were observed for visual stimuli for musically-trained participants than that of musically-untrained participants. Further, the present study also highlights that the overestimation of duration in auditory modality is linked with the length of the musical training. This is an interesting result signifying that musical training cannot simply change the processing of duration rather the length of the training is crucial.

**FIGURE 4:**
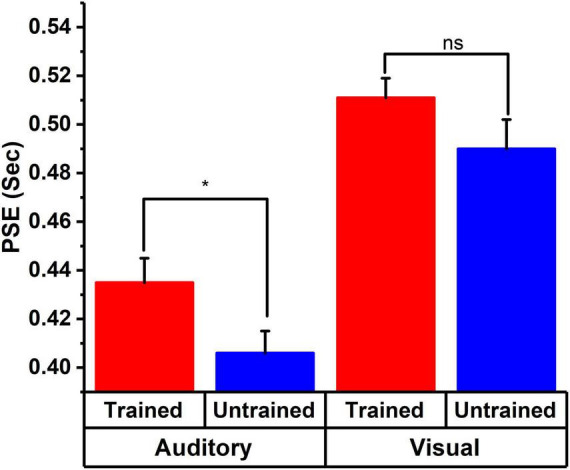
PSE descriptive plot for visual and auditory task shows the PSE for the visual task across different groups. Musically-trained participants’ performance is shown in red, whereas musically-untrained participants’ performance is indicated in blue. The PSE for the visual task for musically-trained and musically-untrained participants is not different. Whereas the PSE for the musically-trained and musically-untrained participants are different for auditory task. *Indicates statistically significant differences (*p* < 0.05).

Previous studies have already observed and demonstrated the modality effect in time perception, indicating that auditory stimuli are perceived to be longer than visual stimuli (Sebel and Wilsoncroft, 1983; Gruber and Block, 2013). It has been argued that stimuli presented in the auditory modality increase arousal levels, leading to the overestimation of subjective duration. A similar effect is observed in our results, suggesting a general overestimation for the auditory modality compared to visual ones. Furthermore, our results indicate differential temporal processing across musically-trained and musically-untrained participants for auditory stimuli (see Figure 4). Specifically, we observed an overestimation of duration for musically-untrained participants compared to musically-trained ones. However, this result is inconsistent with previous findings (Yee et al., 1994; Jones and Yee, 1997; Rammsayer and Altenmüller, 2006). More specifically, previous studies have demonstrated that individuals with musical training exhibit overestimation of temporal information compared to their musically-untrained counterparts (Besson et al., 2011). Examining the counterintuitive results, we probed further and showed that the length of musical training is negatively associated with the perceived duration of auditory stimuli. The longer the musical training, the higher the overestimation of duration. This raises a particularly interesting question about what defines a musician. Are two years of musical training sufficient to bring structural/functional changes in the brain? It could be possible that the two years of musical training may not be sufficient to induce structural/functional changes in the brain that can potentially alter temporal processing. However, as the length of the training increases, it may induce plasticity in the brain and result in changes related to auditory, somatosensory, and motor regions (Bangert and Altenmüller, 2003) and these changes may modulate the processing of time. Additionally, musicians’ better performance in temporal discrimination tasks can be traced to the concepts of neuroplastic adaptation to attentive auditory processing (Rammsayer and Altenmüller, 2006). Also, increment in years of training leads to an increase in gray matter volume in cerebellum (Gaser and Schlaug, 2003). Cerebellum has been found to be involved in cognitive skill learning (Parsons, 2001), music processing (Gaab et al., 2003) and time processing (O’Reilly et al., 2008; Breska and Ivry, 2021). Thus, structural changes in cerebellum may account for differential time perception in musically-trained individuals over the years. Alternatively, the findings from this study can be understood through the lens of enhanced attentional processing in the auditory domain resulting from musical training. Musical training may facilitate improved auditory attention, potentially explaining why musically-trained individuals tend to overestimate time as their training years increase. This interpretation aligns with the attentional gate model of time perception, which posits that enhanced attention modulates the gate’s latency, accumulating more pulses leading to a subjective lengthening of time (Block and Zakay, 1996). Similar mechanisms appear to be operational here. Therefore, as the years of training increase, greater attentional resources are allocated to auditory temporal information. Consequently, as the number of training years increases, the tendency of musically-trained individuals to overestimate the duration also increases.

In contrast to the results obtained from the auditory modality, we did not observe a significant difference between the groups on the visual temporal task. We speculate that this result may arise from the absence of differential allocation of attentional resources between the two groups. Given that musically-trained individuals primarily received training in the auditory modality, it is plausible that they exhibit enhanced attentional engagement for processing auditory stimuli compared to visual stimuli. Previous research has also shown that musicians and non-musicians do not differ significantly in terms of visual attention (Strait et al., 2010). Instead, musicians demonstrate superior auditory processing abilities, likely attributable to more efficient neural pathways for auditory information processing (Strait et al., 2010). This could potentially elucidate our non-significant findings in the visual temporal judgment task (see Figure 4).

The attentional gate model, proposed by Block and Zakay (1996), offers a theoretical framework for interpreting our findings. According to this model, the modulation of attentional gate latency differs between tasks, influencing the accumulation of pulses in the cognitive counter. Since our groups did not exhibit differential effects on visual temporal processing, it is plausible that the latency of the gate remains unchanged for the visual task, resulting in comparable pulse accumulation and no significant differences in visual temporal processing.

Moreover, previous studies have also indicated that visual memory performance does not significantly differ between musicians and non-musicians (Brandler and Rammsayer, 2003; Ho et al., 2003). Although musicians demonstrate a larger left planum temporale region associated with better verbal memory, this advantage does not extend to visual memory tasks, as visual memory primarily relies on the right hemisphere (Chan et al., 1998). Given that memory plays a crucial role in tasks requiring time perception, such as the temporal bisection task, participants must retrieve anchor durations to compare it with probe durations. Hence, the lack of differences in visual memory between the groups may explain our insignificant findings in the visual temporal judgment task. Further, we did not find a significant correlation between the PSE of visual modality and the number of years of musical training. Unlike the auditory temporal judgment task, where overestimation increased with the number of years of training, we did not observe a similar trend for the visual task. This suggests that an increase in training years has no discernible impact on the temporal processing of visual stimuli.

While our study provides interesting results regarding musical training and temporal processing, we did not explicitly measure the general ability of participants across the groups. Given that general ability may play a crucial role in modulating the performance of specific groups, future studies should specifically control for general ability when investigating temporal processing differences between musically-trained and musically-untrained groups. Moreover, our study exclusively utilized the temporal bisection task to demonstrate differential temporal processing between musically-trained and untrained participants. To ensure the robustness and generalizability of our findings, we suggest that future studies should consider multiple temporal tasks to explore temporal processing differences between musically-trained and untrained individuals. This approach would enable a comprehensive understanding of temporal processing abilities beyond the limits of the temporal bisection task. Additionally, it is also important to acknowledge that the findings of the present study may be limited to Indian cultural context. Hence, future research should investigate these aspects across diverse cultural settings, accounting for variables such as age and gender to further enhance the generalizability of the results.

## Conclusion

Regardless of participants’ musical training background, our results emphasize a strong modality effect, with auditory stimuli consistently being overestimated compared to visual stimuli. Interestingly, individuals with musical training showed a distinct pattern of underestimating duration relative to those without such training. Although initially it looks counterintuitive, a detailed analysis reveals that the length of musical training significantly impacts temporal processing within the musically-trained group. These findings shed light on the intricate interplay between musical expertise and time perception, emphasizing the need for nuanced exploration in future research.

## Data availability statement

The raw data supporting the conclusions of this article will be made available by the corresponding author on reasonable request.

## Ethics statement

The studies involving humans were approved by the Thapar Institute of Engineering and Technology, Patiala, Punjab. The studies were conducted in accordance with the local legislation and institutional requirements. The participants provided their written informed consent to participate in this study.

## Author contributions

JM: Data curation, Formal analysis, Methodology, Writing – original draft, Writing – review & editing. KJ: Data curation, Formal analysis, Methodology, Writing – original draft, Writing – review & editing. SS: Data curation, Formal analysis, Methodology, Writing – original draft, Writing – review & editing. AS: Conceptualization, Formal analysis, Investigation, Methodology, Project administration, Resources, Supervision, Visualization, Writing – review & editing.
