# Supplementary material for: A matter of time: how musical training affects time perception
Source: Front Neurosci. 2024 Apr 29;18:1364504. doi: 10.3389/fnins.2024.1364504 (PMC11089211; doi:10.3389/fnins.2024.1364504)
Supplement: Supplementary file 1 [file Data_Sheet_1.PDF]

Results

Analysis of Mixed Repeated Measures ANOVA for Musically-Trained and Untrained Groups.

Descriptive Statistics

Descriptive Statistics

	PSE_Auditory		PSE_Visual	
	Musically-Trained	Musically-Untrained	Musically-Trained	Musically-Untrained
Valid	32	32	32	32
Mean	0.435	0.406	0.511	0.490
Std. Deviation	0.058	0.050	0.046	0.067
Shapiro-Wilk	0.969	0.955	0.969	0.972
P-value of Shapiro-Wilk	0.477	0.196	0.473	0.570
Minimum	0.314	0.310	0.400	0.349
Maximum	0.539	0.480	0.584	0.602

Repeated Measures ANOVA

Within Subjects Effects

Cases	Sum of Squares	df	Mean Square	F	p	η^2_p
Modality	0.208	1	0.208	108.324	< .001	0.636
Modality ✻ Group	5.198×10^{-4}	1	5.198×10^{-4}	0.271	0.604	0.004
Residuals	0.119	62	0.002			

Note. Type III Sum of Squares

Between Subjects Effects

Cases	Sum of Squares	df	Mean Square	F	p	η^2_p
Group	0.020	1	0.020	4.620	0.036	0.069
Residuals	0.269	62	0.004			

Note. Type III Sum of Squares

Descriptives

Descriptives

Modality	Group	N	Mean	SD	SE	Coefficient of Variation
Auditory	Musically-Trained	32	0.435	0.058	0.010	0.133
	Musically-Untrained	32	0.406	0.050	0.009	0.123
Visual	Musically-Trained	32	0.511	0.046	0.008	0.091
	Musically-Untrained	32	0.490	0.067	0.012	0.137

Simple Main Effects

Simple Main Effects - Group

Level of Modality	Sum of Squares	df	Mean Square	F	p
Auditory	0.013	1	0.013	4.634	0.035
Visual	0.007	1	0.007	2.111	0.151

Note. Type III Sum of Squares

Bayesian Independent Samples T-Test For Visual Task Across Groups

Bayesian Independent Samples T-Test

	BF_{10}	error %
PSE_Visual	0.621	0.011

Results

Correlation and Independent t-test Analysis for the Musically-Trained Group.

Descriptive Statistics

Descriptive Statistics

	PSE_Auditory		PSE_Visual	
	Above 5 Years	Below 5 Years	Above 5 Years	Below 5 Years
Valid	19	13	19	13
Mean	0.409	0.471	0.513	0.508
Std. Deviation	0.051	0.048	0.047	0.047
Shapiro-Wilk	0.984	0.901	0.955	0.949
P-value of Shapiro-Wilk	0.981	0.137	0.476	0.581
Minimum	0.314	0.372	0.424	0.400
Maximum	0.510	0.539	0.584	0.573

Independent Samples T-Test (Below and Above 5 Years Training)

Independent Samples T-Test

	t	df	p	Cohen's d	SE Cohen's d
PSE_Auditory	−3.454	30	0.002	−1.243	0.413
PSE_Visual	0.267	30	0.791	0.096	0.360

Note. Student's t-test.

Assumption Checks

Test of Normality (Shapiro-Wilk)

		W	p
PSE_Auditory	Above 5 Years	0.984	0.981
	Below 5 Years	0.901	0.137
PSE_Visual	Above 5 Years	0.955	0.476
	Below 5 Years	0.949	0.581

Note. Significant results suggest a deviation from normality.

Test of Equality of Variances (Levene's)

	F	df_1	df_2	p
PSE_Auditory	0.237	1	30	0.630
PSE_Visual	0.040	1	30	0.842

Descriptives

Group Descriptives

	Group	N	Mean	SD	SE	Coefficient of variation
PSE_Auditory	Above 5 Years	19	0.409	0.051	0.012	0.125
	Below 5 Years	13	0.471	0.048	0.013	0.101
PSE_Visual	Above 5 Years	19	0.513	0.047	0.011	0.092
	Below 5 Years	13	0.508	0.047	0.013	0.092

Correlation Analysis For Auditory Modality

Spearman's Correlations

			n	Spearman's rho	p
PSE_Auditory	-	Years of training	32	−0.418	0.017

Correlation Analysis For Visual Modality

Spearman's Correlations

			n	Spearman's rho	p
PSE_Visual	-	Years of training	32	0.006	0.973
